# Testing Normal Means: The Reconcilability of the *P* Value and the Bayesian Evidence

**DOI:** 10.1155/2013/381539

**Published:** 2013-10-30

**Authors:** Yuliang Yin, Junlong Zhao

**Affiliations:** ^1^School of Economics, Beijing Technology and Business University, Beijing 100048, China; ^2^School of Mathematics and System Science, Beihang University, LMIB of the Ministry of Education, Beijing 100083, China

## Abstract

The problem of reconciling the frequentist and Bayesian evidence in testing statistical hypotheses has been extensively studied in the literature. Most of the existing work considers cases without the nuisance parameters which is not the frequently encountered situation since the presence of the nuisance parameters is very common in practice. In this paper, we consider the reconcilability of the Bayesian evidence against the null hypothesis *H*_0_ in terms of the posterior probability of *H*_0_ being true and the frequentist evidence against *H*_0_ in terms of the *P* value in testing normal means where the nuisance parameters are present. The reconcilability of evidence can be obtained both for testing a normal mean and for the Behrens-Fisher problem.

## 1. Introduction

In the problem of testing a statistical hypothesis *H*_0_, a frequentist may give evidence against *H*_0_ by the observed significance level, the *P* value, while a Bayesian may give it by the posterior probability that *H*_0_ is true. Lindley [1] illustrated the possible discrepancy between the Bayesian and the frequentist evidence. The relationship of these two measures of evidence is then extensively studied in the literature. Pratt [2] revealed that the *P* values are usually approximately equal to the posterior probabilities in the one-sided testing problems. Casella and Berger [3] considered testing the one-sided hypothesis for a location parameter and showed that the lower bounds of the posterior probability over some reasonable classes of priors are exactly equal to the corresponding *P* values in many cases. Some important papers which deal with the reconcilability of the Bayesian and frequentist evidence are Bartlett [4], Cox [5], Shafer [6], Berger and Delampady [7], and Berger and Sellke [8].

Although many researches have been carried out to deal with the problem of reconciling the Bayesian and frequentist evidence and some of them show that evidence is reconcilable in several specific situations, most of the existing work assumes that no other unknown parameters are present except the parameters of interest. In fact, we may be confronted with the nuisance parameters in various situations. In the location-scale settings, for example, when the location parameter is unknown, so is the scale parameter, in general.

However, in significance testing of hypotheses with the nuisance parameters, the classical *P* values are typically not available. Tsui and Weerahandi [9], considering testing the one-sided hypothesis of the form

(1) $$\begin{array}{c} H_{0}:\theta \leq c\;\text{versus}\;H_{1}:\theta >c, \end{array}$$

where *θ* is the parameter of interest and *c* is a fixed constant, introduced the concept of the generalized *P* value, which appears to be useful in situations where conventional frequentist approaches do not provide useful solutions.

Tsui and Weerahandi [9] and some later relevant works formulated the generalized *P* values for many specific examples. Hannig et al. [10] provided a general method for constructing the generalized *P* value via fiducial inference.

In this paper, for the one-sided testing situations about normal means where the nuisance parameters are present, we study the reconcilability of the Bayesian evidence and the generalized *P* value. It is shown that, under the conjugate class of prior distributions, the Bayesian evidence and the generalized *P* value are reconcilable both for the problem of testing a normal mean and for the Behrens-Fisher problem.

This paper is organized as follows. In Section 2, we give the main results of the reconcilability of the *P* value and the Bayesian evidence in testing normal means. Some conclusions and discussions are given in Section 3.

## 2. Main Results

In this section, we consider two testing problems in which the nuisance parameters are present. When no efficient classical frequentist evidence is available because of the presence of the nuisance parameters, we formulate the frequentist evidence by the generalized *P* value.

### 2.1. One-Sample Normal Mean

 Let *X*_1_,…, *X*_*n*_ be a random sample from a normal population *N*(*μ*, *σ* ^2^), where both the mean *μ* and the variance *σ* ^2^ are unknown. Consider now the following problem of testing the mean of a normal distribution

(2) $$\begin{array}{c} H_{0}:\mu \leq c\;\text{versus}\;H_{1}:\mu >c, \end{array}$$

where *c* is a fixed constant.

For this testing problem, where the nuisance parameter is present, we can still obtain the classical *P* value as

(3) $$\begin{array}{c} p\left(x\right)=P\left(T_{n-1}\leq \frac{\sqrt{n}\left(c-\bar{x}\right)}{s}\right), \end{array}$$

where *T*_*n*−1_ is a *t*-variable with *n* − 1 degrees of freedom and $\bar{x}$ and *s* ^2^ stand for the observed sample mean and sample variance, respectively.

To derive the Bayesian evidence, we need a prior for the parameters. One reasonable and conventional class of priors for *μ* and *σ* ^2^ is the following conjugate class of prior distributions *G*_*c*_1__:

(4) $$\begin{array}{c} \mu \;|\;\sigma^{2}\sim N\left(\mu_{0},\frac{\sigma^{2}}{\kappa_{0}}\right),\;\;\;\;\;\;\;\frac{1}{\sigma^{2}}\sim \text{Gamma}\left(\frac{\nu_{0}}{2},\frac{\nu_{0}\sigma_{0}^{2}}{2}\right), \end{array}$$

where the prior parameters (*μ*_0_, *κ*_0_) can be interpreted as the mean and sample size of the normal prior observations and (*σ*_0_^2^, *ν*_0_) the sample variance and sample size of the Gamma prior observations.

Under (4) we have

(5) $$\begin{array}{c} \mu \;|\;x,\sigma^{2}\sim N\left(\mu_{n}\left(x\right),\frac{\sigma^{2}}{\kappa_{n}}\right),\\ \frac{1}{\sigma^{2}}\;|\;x\sim \text{Gamma}\left(\frac{\nu_{n}}{2},\frac{\nu_{n}\sigma_{n}^{2}\left(x\right)}{2}\right), \end{array}$$

where *x* = (*x*_1_,…, *x*_*n*_)′,  *κ*_*n*_ = *κ*_0_ + *n*,  $\mu_{n}(x)=(\kappa_{0}\mu_{0}+n\bar{x})/\kappa_{n}$, *ν*_*n*_ = *ν*_0_ + *n*, and $\sigma_{n}^{2}(x)=[\nu_{0}\sigma_{0}^{2}+(n-1)s^{2}+\kappa_{0}n{\left(\bar{x}-\mu_{0}\right)}^{2}/\kappa_{n}]/\nu_{n}$. Therefore, we can give the posterior density for (*μ*, *σ* ^2^) as

(6) π(μ,σ2 ∣ x)=κn2πσexp⁡[−κn(μ−μn(x))22] ×((νn/2)σn2(x))νn/2Γ(νn/2)σνn+2exp⁡[−νnσn2(x)2σ2]=κn((νn/2)σn2(x))νn/22πΓ(νn/2)σνn+3 ×exp⁡[−κn(μ−μn(x))2+νnσn2(x)2σ2].

Then the marginal posterior density for *μ* can be obtained by integrating out *σ* ^2^ as

(7) π(μ ∣ x)=κn((νn/2)σn2(x))−1/2Γ((νn+1)/2)Γ(νn/2) ×[1+κn(μ−μn(x))2νnσn2(x)]−(νn+1)/2,

from which we know that

(8) $$\begin{array}{c} \frac{\sqrt{\kappa_{n}}\left(\mu -\mu_{n}\left(x\right)\right)}{\sigma_{n}\left(x\right)}\sim t\left(\nu_{n}\right). \end{array}$$

Consequently, the posterior probability of *H*_0_ being true is

(9) $$\begin{array}{c} P\left(H_{0}\;|\;x\right)=P\left(T_{\nu_{n}}\leq \sqrt{\frac{\kappa_{n}}{\sigma_{n}^{2}\left(x\right)}}\left(c-\mu_{n}\left(x\right)\right)\right), \end{array}$$

where *T*_*ν*_*n*__ is a *t*-variable with *ν*_*n*_ degrees of freedom. Notice that if *μ*_0_ = *c*, we have

(10) $$\begin{array}{c} \underset{\kappa_{0},\sigma_{0}\to 0}{\lim}P\left(H_{0}\;|\;x\right)=P\left(T_{\nu_{n}}\leq \sqrt{\frac{\nu_{n}}{n-1}}\frac{\sqrt{n}\left(c-\bar{x}\right)}{s}\right). \end{array}$$

**Lemma 1**

Let *T* be a *t*-variable with *α* degrees of freedom, where *α* is a positive real number. Then for $R=T/\sqrt{\alpha }$ and a fixed constant *r*, *P*(*R* ≤ *r*) is nonincreasing in *α* if *r* ≤ 0 and is nondecreasing in *α* if *r* > 0.

**Proof**

Suppose that *X* is a nonpositive random variable obtained by the negative part of *T*; that is, the density of *X* is

(11) $$\begin{array}{c} f\left(x,\alpha \right)=\frac{2\Gamma \left(\left(\alpha +1\right)/2\right)}{\sqrt{\alpha \pi }\Gamma \left(\alpha /2\right)}{\left(1+\frac{x^{2}}{\alpha }\right)}^{-(\alpha +1)/2},\;x\leq 0. \end{array}$$

Then the density of $Y=X/\sqrt{\alpha }$ is

(12) $$\begin{array}{c} p\left(y,\alpha \right)=\frac{2\Gamma \left(\left(\alpha +1\right)/2\right)}{\sqrt{\pi }\Gamma \left(\alpha /2\right)}{\left(1+y^{2}\right)}^{-(\alpha +1)/2},\;y\leq 0. \end{array}$$

By Theorem 3.3.2 in Lehmann [11], for any fixed nonpositive constant *r*, we have that *P*(*Y* ≤ *r*) is nonincreasing in *α* since it can be verified that the family of densities *p*(*y*, *α*) has monotone likelihood ratio in *y*. This implies that Lemma 1 holds for the case when *r* ≤ 0 since *P*(*R* ≤ *r*) = *P*(*X* ≤ *r*)/2. Since when *r* > 0, we have *P*(*R* ≤ *r*) = 1/2 + *P*(0 ≤ *R* ≤ *r*), the proof for the latter case is completely analogous if we introduce a nonnegative random variable obtained by the positive part of *T*.

Now take $r=\sqrt{n}(c-\bar{x})/(\sqrt{n-1}s)$. By Lemma 1, for *r* ≤ 0, we have

(13) P(Tνnνn≤r)≤P(Tn−1n−1≤r)=P(Tn−1≤n(c−x¯)s).

Then comparing (3) and (10), for *μ*_0_ = *c* and any fixed nonnegative *ν*_0_, we have

(14) $$\begin{array}{c} \underset{\kappa_{0},\sigma_{0}\to 0}{\lim}P\left(H_{0}\;|\;x\right)<p\left(x\right),\;\text{as}\;p\left(x\right)<\frac{1}{2}, \end{array}$$

which implies that

(15) $$\begin{array}{c} \underset{\pi \in G_{c_{1}}}{\inf}P\left(H_{0}\;|\;x\right)<p\left(x\right),\;\text{as}\;p\left(x\right)<\frac{1}{2}. \end{array}$$

The reconcilability of the Bayesian and frequentist evidence is therefore obtained in this testing problem. We summarize this as the following theorem.

**Theorem 2**

For testing the hypothesis of the form (2) under a normal distribution *N*(*μ*, *σ* ^2^) with *σ* ^2^ unknown, the Bayesian and frequentist lines of evidence are reconcilable under the conjugate class of priors (4).

### 2.2. Behrens-Fisher Problem

Now we turn to consider the Behrens-Fisher problem. It is a classical testing situation in which the nuisance parameters are present and no useful pivotal quantities are available. Suppose that *X*_1_,…, *X*_*m*_ and *Y*_1_,…, *Y*_*n*_ are two independent random samples from two normal populations *N*(*μ*_1_, *σ*_1_^2^) and *N*(*μ*_2_, *σ*_2_^2^), respectively, where both *σ*_1_^2^ and *σ*_2_^2^ are completely unspecified. We are interested in testing the hypothesis of the form

(16) $$\begin{array}{c} H_{0}:\mu_{1}-\mu_{2}\leq c\;\text{versus}\;H_{1}:\mu_{1}-\mu_{2}>c, \end{array}$$

where *c* is a fixed constant.

In situations where the traditional frequentist approaches fail to provide useful solutions, the conception of the generalized *P* values introduced by Tsui and Weerahandi [9] appears to be helpful in deriving the frequentist evidence for testing a statistical hypothesis. For this specific problem of testing hypothesis (16), we can give the generalized *P* value as

(17) p(x)=P(x¯−y¯−(m−1s1Umχm−12−n−1s2Vnχn−12)≤c)=P(s2nTn−1−s1mTm−1≤c−(x¯−y¯)),

where *U* ~ *N*(0,1), *V* ~ *N*(0,1), *χ*_*m*−1_^2^ ~ *χ* ^2^(*m* − 1), *χ*_*n*−1_^2^ ~ *χ* ^2^(*n* − 1), *T*_*m*−1_ ~ *t*(*m* − 1), and *T*_*n*−1_ ~ *t*(*n* − 1).

In this problem, we consider the reconcilability of evidence under the following conjugate class of prior distributions *G*_*c*_2__:

(18) $$\begin{array}{c} \mu_{1}\;|\;\sigma_{1}^{2}\sim N\left(\mu_{01},\frac{\sigma_{01}^{2}}{\kappa_{01}}\right),\;\;\frac{1}{\sigma_{1}^{2}}\sim \text{Gamma}\left(\frac{\nu_{01}}{2},\frac{\nu_{01}\sigma_{01}^{2}}{2}\right);\\ \mu_{2}\;|\;\sigma_{2}^{2}\sim N\left(\mu_{02},\frac{\sigma_{02}^{2}}{\kappa_{02}}\right),\;\;\frac{1}{\sigma_{2}^{2}}\sim \text{Gamma}\left(\frac{\nu_{02}}{2},\frac{\nu_{02}\sigma_{02}^{2}}{2}\right). \end{array}$$

Under *G*_*c*_2__, the posterior density of (*μ*_1_, *μ*_2_, *σ*_1_^2^, *σ*_2_^2^) is

(19) π(μ1,μ2,σ12,σ22 ∣ x,y) =f(μ1,μ2,σ12,σ22,x,y)∫−∞∞∫−∞∞∫0∞∫0∞f(μ1,μ2,σ12,σ22,x,y)dσ12 dσ22 dμ1 dμ2,

where

(20) f(μ1,μ2,σ12,σ22,x,y) =(exp⁡[−Σi=1m(xi−μ1)2+κ01(μ1−μ01)2+ν01σ0122σ12      −Σi=1n(yi−μ2)2+κ02(μ2−μ02)2+ν02σ0222σ22])  ×((σ12)(m+ν01+4)/2(σ22)(n+ν02+4)/2)−1.

Let *θ* = *μ*_1_ − *μ*_2_. Then the posterior density of (*θ*, *μ*_2_, *σ*_1_^2^, *σ*_2_^2^) is

(21) π(θ,μ2,σ12,σ22 ∣ x,y) =f(θ,μ2,σ12,σ22,x,y)∫−∞∞∫−∞∞∫0∞∫0∞f(μ1,μ2,σ12,σ22,x,y)dσ12 dσ22 dμ1dμ2,

where

(22) f(θ,μ2,σ12,σ22,x,y) =(exp⁡[−Σi=1m(xi−θ−μ2)2+κ01(θ+μ2−μ01)2+ν01σ0122σ12     −Σi=1n(yi−μ2)2+κ02(μ2−μ02)2+ν02σ0222σ22])   ×((σ12)(m+ν01+4)/2(σ22)(n+ν02+4)/2)−1.

So that the posterior probability of *H*_0_ is

(23) P(H0 ∣ x) =∫−∞c∫−∞∞∫0∞∫0∞f(θ,μ2,σ12,σ22,x,y)dσ12dσ22dμ2dθ∫−∞∞∫−∞∞∫0∞∫0∞f(μ1,μ2,σ12,σ22,x,y)dσ12dσ22dμ1dμ2.

It is straightforward to check that

(24) lim⁡κ01,κ02,σ01,σ02→0P(H0 ∣ x)=Γ((m+ν01+2)/2)Γ((n+ν02+2)/2)π(m−1)(n−1)Γ((m+ν01+1)/2)Γ((n+ν02+1)/2)   ×∫−∞∞∫m(x¯−y¯−c)/s1+ms2r/s1∞(1+t2m−1)(−m−ν01−2)/2        ×(1+r2n−1)(−n−ν02−2)/2dt dr,  =P(x¯−y¯−(m+ν01+1s1Um+ν02+2χm+ν01+12     −n+ν02+1s2Vn+ν02+2χm+ν01+12)≤c)  =P(s2n+ν02+2Tn+ν02+1−s1m+ν01+2Tm+ν01+1     ≤c−(x¯−y¯)),

where $\bar{x}$ and $\bar{y}$ are the observation of the sample mean $\bar{X}$ and $\bar{Y}$, respectively, *s*_1_^2^ and *s*_2_^2^ are that of the sample variance *S*_1_^2^ and *S*_2_^2^ respectively, *U* ~ *N*(0,1), *V* ~ *N*(0,1), *χ*_*m*+*ν*_01_+1_^2^ ~ *χ* ^2^(*m* + *ν*_01_ + 1) and *χ*_*n*+*ν*_02_+1_^2^ ~ *χ* ^2^(*n* + *ν*_02_ + 1).

Now we prove an interesting result that, when *m* and *n* are sufficiently large, the frequentist and Bayesian lines of evidence given respectively by (17) and (23) are reconcilable for any fixed *ν*_01_ and *ν*_02_ under the prior class of *G*_*c*_2__.

**Theorem 3**

As min⁡{*m*, *n*}→+*∞*, for any fixed 0 < *ν*_01_, *ν*_02_ < *∞*, we have

(25) $$\begin{array}{c} \underset{\kappa_{01},\kappa_{02},\sigma_{01},\sigma_{02}\to 0}{\lim}P\left(H_{0}\;|\;x\right)<p\left(x\right),\;as\;p\left(x\right)<\frac{1}{2}, \end{array}$$

which implies that

(26) $$\begin{array}{c} \underset{\pi \in G_{c_{2}}}{\inf}P\left(H_{0}\;|\;x\right)<p\left(x\right),\;as\;p\left(x\right)<\frac{1}{2}. \end{array}$$

**Proof**

Let

(27) $$\begin{array}{c} A_{1}=\frac{\left(n-1\right)s_{2}^{2}}{n\chi_{n-1}^{2}},\\ B_{1}=\frac{\left(n+\nu_{02}+1\right)s_{2}^{2}}{\left(n+\nu_{02}+2\right)\chi_{n+\nu_{02}+1}^{2}}. \end{array}$$

(I) We first prove that, given *s*_2_, as *n* is sufficiently large, we have

(28) $$\begin{array}{c} B_{1}\overset{d}{<}A_{1}. \end{array}$$

In fact, for any *γ* > 0, as *n* is sufficiently large,

(29) P(B1<γ)=P(χn+ν02+12>(n+ν02+1)s22(n+ν02+2)γ),=P(χn+ν02+12>(n−1)s22nγ+ϵ(n,γ)),

where *ϵ*(*n*, *γ*) = (*ν*_02_ + 2)*s*_2_^2^/[(*n* + *ν*_02_ + 2)*nγ*] → 0, as *n* → *∞*. On the other hand, we have

(30) $$\begin{array}{c} P\left(A_{1}<\gamma \right)=P\left(\chi_{n-1}^{2}>\frac{\left(n-1\right)s_{2}^{2}}{n\gamma }\right). \end{array}$$

Since $\chi_{n+\nu_{02}+1}^{2}\overset{d}{>}\chi_{n-1}^{2}$ holds for any *n*, it follows that, as *n* → *∞*,

(31) $$\begin{array}{c} P\left(B_{1}<\gamma \right)>P\left(A_{1}<\gamma \right). \end{array}$$

That is,

(32) $$\begin{array}{c} B_{1}\overset{d}{<}A_{1}. \end{array}$$

Similarly, as *m* → +*∞*, we have

(33) $$\begin{array}{c} B_{2}:=\frac{\left(m+\nu_{01}+1\right)s_{1}^{2}}{\left(m+\nu_{01}+2\right)\chi_{m+\nu_{01}+1}^{2}}\overset{d}{<}\frac{\left(m-1\right)s_{1}^{2}}{m\chi_{m-1}^{2}}:=A_{2}. \end{array}$$

Therefore, we have

(34) $$\begin{array}{c} B_{1}+B_{2}\overset{d}{<}A_{1}+A_{2}. \end{array}$$

Consequently, we have

(35) $$\begin{array}{c} \sqrt{B_{1}+B_{2}}\overset{d}{<}\sqrt{A_{1}+A_{2}}. \end{array}$$

(II) We now show that the final conclusion holds. In fact, if we let $C(x,y)=c-(\bar{x}-\bar{y})$, then

(36) p(x)=P(A1V−A2U≤C(x,y))=E(P(A1V−A2U≤C(x,y) ∣ A1,A2))=E(Φ(C(x,y)A1+A2)),

where Φ(·) stands for the cumulative distribution function of a standard normal distribution and the last equation is due to the fact that *U* and *V* are independent normal distributions.

Similarly, for (24), we have

(37) lim⁡κ01,κ02,σ01,σ02→0P(H0 ∣ x,y)  =E(P(B1V−B2U≤C(x,y) ∣ B1,B2))  =E(Φ(C(x,y)B1+B2)).

Note that for each *C*(*x*, *y*) in (−*∞*, 0), Φ(*C*(*x*, *y*)/*R*) is increasing in *R* ∈ (0, *∞*). Therefore, by (35), we have

(38) $$\begin{array}{c} \Phi \left(\frac{C\left(x,y\right)}{\sqrt{B_{1}+B_{2}}}\right)\overset{d}{<}\Phi \left(\frac{C\left(x,y\right)}{\sqrt{A_{1}+A_{2}}}\right),\;\text{as}\;C\left(x,y\right)<0. \end{array}$$

Consequently, we have

(39) E(Φ(C(x,y)B1+B2)<E(Φ(C(x,y)A1+A2))),as  C(x,y)<0.

In addition, by the symmetry of the *t*-distribution, it follows that *C*(*x*, *y*) < 0 is equivalent to *p*(*x*) < 1/2. Therefore, by (36), (37), and (39), the conclusion of Theorem 3 holds.

The following theorem shows that, even for fixed *m* and *n* with 2 < *m*, *n* < *∞*, we still obtain the reconcilability of the frequentist and Bayesian evidence.

**Theorem 4**

As min⁡{*ν*_01_, *ν*_02_} → *∞*, the conclusion of Theorem 3 holds for any fixed *m* and *n* with 2 < *m*, *n* < *∞*; that is,

(40) $$\begin{array}{c} \underset{\pi \in G_{c_{2}}}{\inf}P\left(H_{0}\;|\;x\right)<p\left(x\right),\;as\;p\left(x\right)<\frac{1}{2}. \end{array}$$

**Proof**

We still adopt the notations of Theorem 3. We first prove that, as *ν*_02_ → *∞*,

(41) $$\begin{array}{c} B_{1}\overset{d}{<}A_{1}. \end{array}$$

By the proof of Theorem 3, we have

(42) $$\begin{array}{c} P\left(B_{1}<\gamma \right)=P\left(\chi_{n+\nu_{02}+1}^{2}>\frac{\left(n-1\right)s_{2}^{2}}{n\gamma }+\epsilon \left(n,\gamma \right)\right), \end{array}$$

where

(43) $$\begin{array}{c} \epsilon \left(n,\gamma \right)=\frac{\left(\nu_{02}+2\right)s_{2}^{2}}{\left(n+\nu_{02}+2\right)n\gamma }. \end{array}$$

It is obvious that

(44) $$\begin{array}{c} \underset{\nu_{02}}{\sup}\;\epsilon \left(n,\gamma \right)=\underset{\nu_{02}\to \infty }{\lim}\epsilon \left(n,\gamma \right)=\frac{s_{2}^{2}}{n\gamma }. \end{array}$$

Let *f*_*n*_(*t*) denote the density function of *χ*_*n*_^2^. Then it is easy to see that *f*_*n*_(*t*) reaches the maximum at *t* = *n* − 2 and that max⁡_*t*_ *f*_*n*_(*t*) = *f*_*n*_(*n* − 2) → 0, as *n* → *∞*. Therefore, as *ν*_02_ is sufficiently large, it holds that

(45) P(B1<γ)=P(χn+ν02+12>s22γ)+ϵ(ν02)=P(B~1<γ)+ϵ(ν02)

for some *ϵ*(*ν*_02_) > 0, where *ϵ*(*ν*_02_) → 0, as *ν*_02_ → *∞*, and ${\tilde{B}}_{1}=s_{2}^{2}/\chi_{n+\nu_{02}+1}^{2}$.

Therefore, for any fixed *n*  (2 < *n* < *∞*), as *ν*_02_ → *∞*, we have

(46) P(B~1<γ)=P(χn+ν02+12>s22γ)=P(χn+ν02+12−(n+ν02+1)2(n+ν02+1)   >s22/γ−(n+ν02+1)2(n+ν02+1))=1−Φ(s22/γ−(n+ν02+1)2(n+ν02+1)) −ϵ1(ν02)→1,

where *ϵ*_1_(*ν*_02_) → 0, as *ν*_02_ → *∞*.

On the other hand, for any fixed *n*  (2 < *n* < *∞*) and *γ* ≠ 0, we have

(47) $$\begin{array}{c} P\left(A_{1}<\gamma \right)=P\left(\chi_{n-1}^{2}>\frac{\left(n-1\right)s_{2}^{2}}{n\gamma }\right)\leq 1-\epsilon_{0}, \end{array}$$

for some *ϵ*_0_ > 0. Therefore, as *ν*_02_ → *∞*, by (46) and (47), we have $P\left({\tilde{B}}_{1}<\gamma \right)-P(A_{1}<\gamma )>0$. Furthermore, by (45), we have

(48) $$\begin{array}{c} P\left(B_{1}<\gamma \right)>P\left(A_{1}<\gamma \right). \end{array}$$

That is,

(49) $$\begin{array}{c} B_{1}\overset{d}{<}A_{1}. \end{array}$$

Similarly, for any fixed *m*  (2 < *m* < *∞*), as *ν*_01_ → *∞*, we have

(50) $$\begin{array}{c} B_{2}\overset{d}{<}A_{2}. \end{array}$$

Therefore, similar to Theorem 3, as min⁡{*ν*_01_, *ν*_02_} → *∞*, for any fixed *m* and *n* with 2 < *m*, *n* < *∞*, we have

(51) $$\begin{array}{c} \sqrt{B_{1}+B_{2}}\overset{d}{<}\sqrt{A_{1}+A_{2}}. \end{array}$$

The rest part of the proof is similar to that of (II) of Theorem 3.

The following simulation results show that even for small and fixed values of *m* and *n* or *ν*_01_ and *ν*_02_, the generalized *P* value and Bayesian evidence for testing the Behrens-Fisher problem are still reconcilable.

For fixed *ν*_01_ = 0.5 and *ν*_02_ = 1 and for *s*_1_^2^ = 1 and *s*_2_^2^ = 4, taking different values of *m* and *n*, some results of comparing the *P* value and lim⁡_*κ*_01_,*κ*_02_,*σ*_01_,*σ*_02_→0_ *P*(*H*_0_ | *x*) are listed in Table 1.

For fixed *m* = 8 and *n* = 10 and for *s*_1_^2^ = 1 and *s*_2_^2^ = 4, taking different values of *ν*_01_ and *ν*_02_, we list some results of comparing *P* value and lim⁡_*κ*_01_,*κ*_02_,*σ*_01_,*σ*_02_→0_ *P*(*H*_0_ | *x*) in Table 2.

## 3. Conclusions

In the presence of the nuisance parameters, we study the reconcilability of the *P* value and the Bayesian evidence in the one-sided hypothesis testing problem about normal means. For the problem of testing a normal mean where the nuisance parameter is present, it is shown that the Bayesian and frequentist lines of evidence are reconcilable. For the Behrens-Fisher problem, it is illustrated that if the sample sizes *m* and *n* tend to infinity, then for fixed prior parameters *ν*_01_ and *ν*_02_, both lines of evidence are reconcilable. Furthermore, it is illustrated that if the prior parameters *ν*_01_ and *ν*_02_ tend to infinity, then for any fixed sample sizes *m* and *n*, lines of evidence are reconcilable. Simulation results show that even for small and fixed values of sample sizes *m* and *n* or for small values of prior parameters *ν*_01_ and *ν*_02_, the reconcilable conclusion of the Bayesian and frequentist evidence still holds.

This provides another illustration of testing situation where the Bayesian and frequentist evidence can be reconciled and may therefore to some extent prevent people from debasing or even dismissing *P* values as evidence in hypothesis testing problems. Furthermore, our results of the reconcilability in the one-sided testing situations may help us to come to the idea that maybe it is arbitrary to assert the irreconcilability of the evidence in the two-sided (point or interval) hypothesis testing problems and perhaps we should be concerned more about the appropriateness of the methods we employ to tackle a two-sided hypothesis in both the frequentist and the Bayesian frameworks.

## Figures and Tables

**Table 1:** *P* value and lim⁡*P*(*H*_0_ | *x*) for testing the Behrens-Fisher problem for different *m* and *n*.

	*m* = 2, *n* = 2								
$\;c-(\bar{x}-\bar{y})$	−2.5000	−2.1000	−1.8000	−1.6000	−1.3000	−0.9000	−0.5000	−0.3000	−0.1000
*p*(*x*)	0.1165	0.1405	0.1785	0.1880	0.2330	0.3110	0.3850	0.4155	0.4745
lim⁡*P*(*H*_0_ | *x*)	0.0195	0.0420	0.0505	0.0705	0.1280	0.1945	0.3140	0.3770	0.4675

	*m* = 5, *n* = 5								

$c-(\bar{x}-\bar{y})$	−2.5000	−2.1000	−1.8000	−1.6000	−1.3000	−0.9000	−0.5000	−0.3000	−0.1000
*p*(*x*)	0.0385	0.0520	0.0760	0.0995	0.1405	0.2250	0.3460	0.3845	0.4705
lim⁡*P*(*H*_0_ | *x*)	0.0060	0.0195	0.0400	0.0465	0.0765	0.1630	0.2840	0.3640	0.4630

	*m* = 1, *n* = 8								

$c-(\bar{x}-\bar{y})$	−2.5000	−2.1000	−1.8000	−1.6000	−1.3000	−0.9000	−0.5000	−0.3000	−0.1000
*p*(*x*)	0.1165	0.1405	0.1785	0.1880	0.2330	0.3110	0.3850	0.4155	0.4745
lim⁡*P*(*H*_0_ | *x*)	0.0195	0.0420	0.0505	0.0705	0.1280	0.1945	0.3140	0.3770	0.4675

	*m* = 3, *n* = 5								

$c-(\bar{x}-\bar{y})$	−2.5000	−2.1000	−1.8000	−1.6000	−1.3000	−0.9000	−0.5000	−0.3000	−0.1000
*p*(*x*)	0.0600	0.0755	0.1030	0.1360	0.1765	0.2615	0.3495	0.4355	0.4595
lim⁡*P*(*H*_0_ | *x*)	0.0070	0.0205	0.0405	0.0545	0.0845	0.1715	0.3005	0.3760	0.4670

	*m* = 12, *n* = 3								

$c-(\bar{x}-\bar{y})$	−2.5000	−2.1000	−1.8000	−1.6000	−1.3000	−0.9000	−0.5000	−0.3000	−0.1000
*p*(*x*)	0.0835	0.1130	0.1260	0.1585	0.2075	0.2975	0.3700	0.4245	0.4995
lim⁡*P*(*H*_0_ | *x*)	0.0185	0.0315	0.0515	0.0730	0.1025	0.1810	0.2905	0.3635	0.4585

**Table 2:** *P* value and lim⁡*P*(*H*_0_ | *x*) for testing the Behrens-Fisher problem for different *ν*_01_ and *ν*_02_.

	*ν*_01_ = 0.5, *ν*_02_ = 0.5								
$\;c-(\bar{x}-\bar{y})$	−2.5000	−2.1000	−1.8000	−1.6000	−1.3000	−0.9000	−0.5000	−0.3000	−0.1000
*p*(*x*)	0.0030	0.0080	0.0130	0.0240	0.0490	0.1310	0.2720	0.3495	0.4565
lim⁡*P*(*H*_0_ | *x*)	0.0010	0.0035	0.0060	0.0135	0.0280	0.1030	0.2245	0.3375	0.4410

	*ν*_01_ = 2, *ν*_02_ = 2								

$c-(\bar{x}-\bar{y})$	−2.5000	−2.1000	−1.8000	−1.6000	−1.3000	−0.9000	−0.5000	−0.3000	−0.1000
*p*(*x*)	0.0025	0.0045	0.0165	0.0270	0.0545	0.1130	0.2550	0.3480	0.4595
lim⁡*P*(*H*_0_ | *x*)	0.0005	0.0015	0.0050	0.0080	0.0245	0.1000	0.2125	0.3155	0.4345

	*ν*_01_ = 0.2, *ν*_02_ = 0.5								

$c-(\bar{x}-\bar{y})$	−2.5000	−2.1000	−1.8000	−1.6000	−1.3000	−0.9000	−0.5000	−0.3000	−0.1000
*p*(*x*)	0.0025	0.0085	0.0145	0.0295	0.0470	0.1225	0.2550	0.3400	0.4610
lim⁡*P*(*H*_0_ | *x*)	0.0015	0.0030	0.0045	0.0130	0.0275	0.1055	0.2215	0.3245	0.4455

	*ν*_01_ = 0.5, *ν*_02_ = 0.2								

$c-(\bar{x}-\bar{y})$	−2.5000	−2.1000	−1.8000	−1.6000	−1.3000	−0.9000	−0.5000	−0.3000	−0.1000
*p*(*x*)	0.0055	0.0070	0.0170	0.0265	0.0545	0.1215	0.2485	0.3535	0.4650
lim⁡*P*(*H*_0_ | *x*)	0.0010	0.0055	0.0070	0.0125	0.0440	0.1085	0.2500	0.3285	0.4300

	*ν*_01_ = 5, *ν*_02_ = 0.5								

$c-(\bar{x}-\bar{y})$	−2.5000	−2.1000	−1.8000	−1.6000	−1.3000	−0.9000	−0.5000	−0.3000	−0.1000
*p*(*x*)	0.0025	0.0060	0.0150	0.0230	0.0570	0.1145	0.2645	0.3685	0.4460
lim⁡*P*(*H*_0_ | *x*)	0.0020	0.0025	0.0065	0.0095	0.0330	0.0890	0.2205	0.3105	0.4305
